# Sparse Codebook Model of Local Structures for Retrieval of Focal Liver Lesions Using Multiphase Medical Images

**DOI:** 10.1155/2017/1413297

**Published:** 2017-02-13

**Authors:** Jian Wang, Xian-Hua Han, Yingying Xu, Lanfen Lin, Hongjie Hu, Chongwu Jin, Yen-Wei Chen

**Affiliations:** ^1^Graduate School of Information Science and Engineering, Ritsumeikan University, Kusatsu, Japan; ^2^National Institute of Advanced Industrial Science and Technology, Tokyo, Japan; ^3^College of Computer Science and Technology, Zhejiang University, Hangzhou, China; ^4^Department of Radiology, Sir Run Run Shaw Hospital, Hangzhou, China

## Abstract

Characterization and individual trait analysis of the focal liver lesions (FLL) is a challenging task in medical image processing and clinical site. The character analysis of a unconfirmed FLL case would be expected to benefit greatly from the accumulated FLL cases with experts' analysis, which can be achieved by content-based medical image retrieval (CBMIR). CBMIR mainly includes discriminated feature extraction and similarity calculation procedures. Bag-of-Visual-Words (BoVW) (codebook-based model) has been proven to be effective for different classification and retrieval tasks. This study investigates an improved codebook model for the fined-grained medical image representation with the following three advantages: (1) instead of SIFT, we exploit the local patch (structure) as the local descriptor, which can retain all detailed information and is more suitable for the fine-grained medical image applications; (2) in order to more accurately approximate any local descriptor in coding procedure, the sparse coding method, instead of *K*-means algorithm, is employed for codebook learning and coded vector calculation; (3) we evaluate retrieval performance of focal liver lesions (FLL) using multiphase computed tomography (CT) scans, in which the proposed codebook model is separately learned for each phase. The effectiveness of the proposed method is confirmed by our experiments on FLL retrieval.

## 1. Introduction

Liver cancer is one of the leading causes of death worldwide. The development of medical imaging techniques, such as computed tomography (CT) and magnetic resonance imaging (MRI), gives more and more detailed information about the inner structure of human body. This detailed information prospects the possibility of early detection for some types of liver lesions, while early diagnosis and treatment is the most effective way to reduce the liver cancer death. On the other hand, multiphase contrast enhanced CT scan is generally employed as the primary technique for detection and characterization of liver lesions, due to the clinical observation that different types of liver lesions exhibit different visual characteristics at various time points after intravenous contrast injection. In multiphase contrast enhanced CT scan procedure, the transition of visual features over time is captured from CTs scanned before and after contrast injection. The noncontrast enhanced (NC) phase image is obtained from scans before injection and three more phases' images are scanned at different times after contrast injection, including arterial (ART) phase, portal venous (PV) phase, and delayed (DL) phase, scanned at 25~40 s, 60~75 s, and 3~5 minutes after contrast injection, respectively. Due to the development of medical imaging techniques and the availability of the multiphase CT scans, medical volumes have become of higher and higher resolution and larger-scale, which prospects more accurate diagnosis while simultaneously leading to heavy burden to radiologists and medical experts for interpreting the CT images. On the other hand, with the ICT technology, it also becomes potential for medical experts to share more and more medical data and the corresponding diagnosis and treatment experiences. However, with the accumulated large-scale medical data with experienced clinical cases, how to search the most similar cases to a query sample (only medical image) for assistance in making decisions of diagnosis and treatment becomes an important and challenging task in both academic and clinical sites. This study concentrates on the similar case retrieval of focal liver lesions to a CT image query with multiphase CT scans.

Characterization of liver lesions is gaining more and more research attention recently, where most work focused on the discriminated feature representation of the medical volume images. In [1], a CBIR system was proposed for retrieving the similar cases for three types of liver lesions by using global features and local features based on image density and texture. In [2], two attributes of margin sharpness extracted from sigmoid curves, including the intensity difference between a lesion and its surroundings, and the sharpness of the intensity transition across the lesion boundary are used for distinguishing different types of lesions. The method was evaluated in both simulations and CT scans of liver and lung lesions and competitive results were achieved. To make use of contrast enhancement features of CT scans, various methods were proposed on multiphase medical images. Quatrehomme et al. proposed to perform classification using visual features and law measures on multiphase CT images to classify five types of liver lesions. Roy et al. [3] used 4 kinds of features, including density, temporal density, texture, and temporal texture, which are derived from four-phase medical images to retrieve the most similar images in five types of liver lesions. The texture features are six coefficients computed from 3D gray-level cooccurrence matrix (GLCM). What is more, the tumors are partitioned into three subvolumes to capture spatial information and the algorithm is accelerated by performing parallel computing on the three subvolumes. Although the related work manifests the potential of retrieving the similar cases of a query FLL input, most of them only exploited low-level features such as intensity, texture, and simple shape descriptors, and thus there still is large space to improve the retrieval performance for real applications. The Bag-of-Visual-Words (BoVW) method has been successfully applied to many natural image classification and retrieval tasks [4–8]. Derived from a few representative triple-phase slices, the Bag-of-Visual-Words (BoVW) with SIFT local features, intensity, texture, and shape-based features is used to distinguish three lesion types [9]. Yang et al. [10] has also used BoVW for retrieval of focal liver lesions in three types. Recently, Diamant et al. [11] presented an improved automated liver lesion classification method using single-phase (PV) medical images based on* K*-means clustering technique. All the above-mentioned methods exploited low-level feature or BoVW-based middle-level features for retrieval or classification of different types of FLLs and have proven the potential capability to FLL characterization and clinical applications. Therein, the middle-level feature, BoVW, presented the promising retrieval performance of FLLs [9–11] compared to low-level features. However the widely used BoVW-based model for medical image representation basically (1) applies the handcrafted SIFT as local features, which would lead to some tiny structure loss and is unsuitable for the fine-grained medical images; (2) uses* K*-means for codebook learning and coded vector calculation, which approximates any local descriptor using one learned visual word only and leads to large reconstruction error; (3) extracts middle-level feature using one-phase data only, which may be inapplicable for some types of FLL cases. Therefore, there still is large space for improving the performance for FLL characterization and representation.

**Figure 1:**
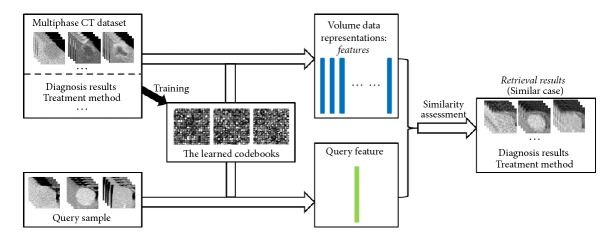
The proposed CBMIR system for FLLs retrieval.

In this study, we explore an improved sparse codebook-based feature representation for multiphase CT volume images and apply the extracted middle-level features for retrieving FLLs to assist diagnosis decision-making of different types of FLLs. The proposed FLL retrieval system is shown in Figure 1, where we concentrate on the discriminated feature extraction from multiphase CT volumes using the improved codebook model. In the proposed codebook model, instead of using SIFT as the local descriptors, which are calculated as the histograms of the quantized orientation and can effectively represent the distinguished part of a specific object in general images, we directly exploit the local patch as the local descriptor, which can retain all detailed information and is more suitable for the fine-grained medical image application. Furthermore, in order to more accurately approximate any local descriptor in coding procedure, the sparse coding method, instead of* K*-means algorithm, is employed to overcomplete codebook learning. Experiments on five types of FLL retrieval show that the proposed feature extraction method can achieve promising retrieval performance compared with state-of-the-art methods.

The rest of this paper is organized as follows.  Section 2 introduces the proposed codebook model for multiphase medical image representation and the used coding strategy: sparse coding for more accurate approximation of any local descriptor.  Section 3 talks about the dataset, experiments settings, and experimental results with different parameters. Finally, we conclude the paper in Section 4.

## 2. The Sparse Codebook Model of Local Structures for Medical Image Representation

**Figure 2:**
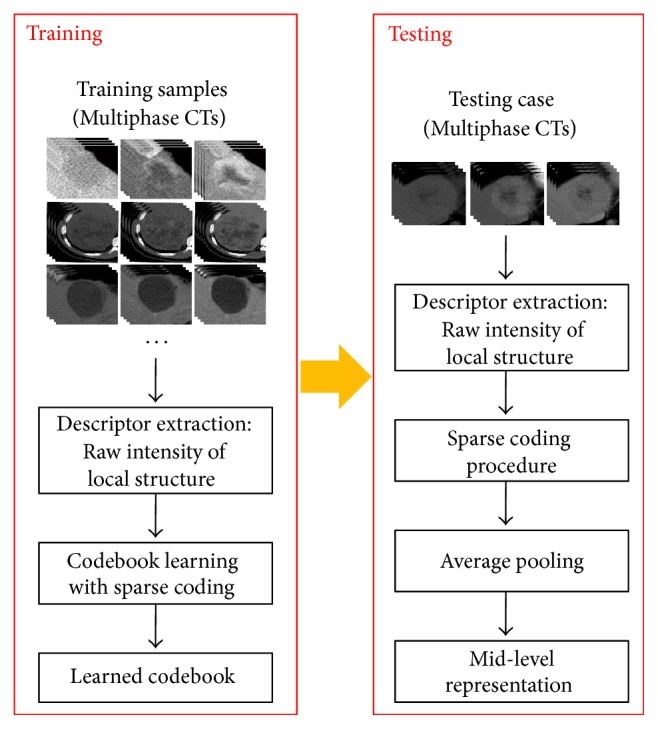
Proposed sparse codebook model with local structures.

Typically, the codebook model, BoVW representation, involves four major steps: (1) key-point detection for interested image regions; (2) local descriptor extraction for representing the interested image regions around key points; (3) coding via quantizing the local descriptors in terms of a predefined dictionary (also called codebook), which generally learned from a large amount of prepared local descriptors with* K*-means algorithm; (4) pooling operation by accumulating the coded feature vectors into a fixed-length representation feature of an image. The local descriptors in the conventional BoVW model generally employed SIFT, which is calculated as the histogram of the quantized orientation and can effectively represent the distinguished part of a specific object in general images. However, this study concentrates on extraction of middle-level features for the fine-grained medical FLL data, and the rough quantization of orientation in SIFT would lead to a lot of detailed information loss, which may not orient the effective representation of the interested image regions for a medical FLL image. Furthermore, the conventional BoVW model used* K*-means algorithm to learn the codebook (set of visual words) and approximated any local descriptor with only one visual word, which would lead to large reconstruction error. Therefore, this study proposes a sparse codebook model oriented for fine-grained FLL image representation, where the local patch of the interested image region is directly used as the local descriptor for retaining all detailed texture and the sparse coding, instead of* K*-means algorithm, is employed for codebook learning and coding any local descriptors. Furthermore, we also combine the codebook-based feature representations of multiphase CT volumes. The proposed sparse codebook model with local structures is shown in Figure 2.

### 2.1. Local Descriptors for Representing the Interested Image Regions

Recent research [12, 13] in the computer vision field showed that it is possible to discriminate between textures using pixel neighborhoods such as a small patch, an *l* × *l* pixel region. Awate et al. [14] explored nonparametric neighborhood statistics and manifested promising performance for texture segmentation. Pietikäinen et al. [13, 15] showed that despite the global structure of the textures, very good discrimination can be achieved by exploiting the distributions of such pixel neighborhoods. Therefore, the exploitation of these microstructures for representing images in the distributions of local descriptors has gained much attention and has led to state-of-the-art performances [13, 15, 16] for different classification and segmentation problems in computer vision. As we know different types of FLLs mainly manifest different intensity variance, which means that there are different textures for different types of FLLs. Thus, this study explores the local patches (texture structures) as local descriptors in the codebook model.

Given an image *I* and the patch region with size *l* × *l* centered at the *i*th pixel, we directly use intensities, *I*_*i*_^*j*^, *j* = 1,2,…, *l* × *l*, of all pixels in the patch regions as a local descriptor **y**_*i*_ = [*I*_*i*_^1^, *I*_*i*_^2^,…, *I*_*i*_^*l*×*l*^]. The representation of a pixel directly uses the neighboring pixels' intensities, which not only considers the intensity but also retains the variation in intensity (texture) without any detained structure loss and thus would adapt to the fine-grained medial FLL retrieval application. In our study, we take the *l* × *l* local patches of all pixels in a medical image as the local descriptor set for coding.

### 2.2. Codebook Learning Algorithm: From* K*-Means to Sparse Coding

Given a set of prepared local descriptors, **Y** = [**y**_1_, **y**_2_,…, **y**_*N*_], the codebook model firstly learns a small set of visual words (prototype features) for coding any local descriptor. A common strategy for codebook learning in the BoVW model usually applies the* K*-means algorithm. A family of signals **Y** = {**y**_*i*_}_*i*=1_^*N*^ can be represented by the nearest neighbor in a codebook **D** = [**d**_1_, **d**_2_,…, **d**_*K*_], *N* ≫ *K*, in which a codeword **d**_*i*_ is a column vector. The codebook **D** is learned in the* K*-means algorithm by solving the least-square problem as follows:

(1) arg minD,xi ∑i=1Nyi−Dxi2,s.t. xi0=1, xi1=1, xi≥0,∀i,

where ‖·‖_0_ and ‖·‖_1_ refer to *L*_0_-norm and *L*_1_-norm, separately. **X** = [**x**_1_, **x**_2_,…, **x**_*N*_] are the coded vectors for **Y**. Single nonzero entry in each coded vector **x**_*i*_ is ensured by the constraint ‖**x**_*i*_‖_0_ = 1, ∀*i*, and the coding weight is always 1, formulated as the second constraint term: ‖**x**_*i*_‖_1_ = 1, ∀*i*. The* K*-means is widely used in codebook training because of its simplicity. However, it is too restricted to approximate signals properly by allowing only one codeword from the codebook.

As a generalization of the* K*-means algorithm, the sparse coding technique employs a linear combination of codewords for the representation of each signal, which means more than one nonzero entries in coding, and the weights can be calculated to be arbitrary values but not limited to 1. The intuitive way for the sparse coding problem can be formulated to optimize the following objective function:

(2) arg minD,X Y−DX22,s.t. X0≤αK,

where **X** is the sparse approximation of **Y** on codebook **D**. *α* is a sparsity measure, which is a ratio between the number of nonzero entries in **x**_*i*_ and the total number of codewords in **D**. *α∗K* controls the maximum number of codewords that can be used for approximation of the input signal **y**_*i*_.

Similar to realization of* K*-means algorithm, there are two stages: sparse coding stage and codebook update stages, in the codebook learning of sparse coding, as shown in Algorithm 1. In consideration of simplicity and efficiency, we employ the Orthogonal Matching Pursuit (OMP) algorithm for coefficient calculation and* K*-SVD method for codebook updating in the two stages, respectively. The detailed implementation of these two methods is described in the following section. Furthermore, we also implement the alternating direction method of multipliers (ADMM) for solving our sparse coding problem, which is an algorithm to solve convex optimization problems by breaking them into smaller pieces for easier handling. Please refer to the existing work [17] for details.

### 2.3. The Implementation Algorithms: OMP and *K*-SVD in Sparse Coding

#### 2.3.1. OMP for Coefficient Calculation

With the fixed codebook, OMP algorithm is a simple and efficient way to solve the sparse approximation problem, which is NP-hard because of the overcomplete codebook **D**. OMP is an iterative greedy algorithm, in which at each step a column codebook vector is selected, which is most correlated with the current residuals, and then the selected column codebook vector is add to the set of selected visual word set being used for calculating the coded coefficients, which can be effectively solved with the least-square problem due to the smaller number of the selected visual words than the dimension of the input signal **x**_*i*_. The algorithm updates the residuals by projecting the input signal onto the linear subspace spanned by the visual word set that has already been selected and the algorithm then iterates until a predefined stop criterion is satisfied.

#### 2.3.2. *K*-SVD for Codebook Updating

*K*-SVD was proposed for generating a dictionary of spare representation, via singular value decomposition (SVD). It is a generalization of the* K*-means clustering method.* K*-SVD works by iteratively alternating between coefficient calculation of the input data based on the current dictionary and updating the atoms in the dictionary to better fit the data. As introduced above the coefficient calculation is implemented via OMP algorithm; this part mainly describes the atom updating procedure, where each of the codewords will be updated once a time, assuming that all the other codewords and the coded vector **x**_*i*_ are fixed. The update of the* k*th atom is done by rewriting the penalty term as

(3) Y−DX2Y−∑j≠kKdjxjT−dkxkT2=Ek−dkxkT2,

where *K* is the total number of codewords and the row vector **x**_*k*_^*T*^ contains the coefficients of all input signals on codeword **d**_*k*_. **E**_*k*_ represents the reconstruction residuals using all codewords excepting **d**_*k*_. After adding **d**_*k*_ for approximating the input signals, we expect that the reconstruction error in (3) is minimized with the fixed **E**_*k*_, which is formulated as the following formula:

(4) arg mindk,xkT Ek−dkxkT2

which can be easily solved by applying SVD. However, **x**_*k*_^*T*^ can be filled and the sparsity will be destroyed if simply SVD is applied on **E**_*k*_. To address this problem, a constraint function **ω**_*k*_ is defined to remove the zero entries of **x**_*k*_^*T*^ and the corresponding elements in **E**_*k*_, achieving **x**_*k*_^*R*^ and **E**_*k*_^*R*^, respectively. Applying SVD on **E**_*k*_^*R*^, **E**_*k*_^*R*^ = **U**Δ**V**^*T*^, the codeword **d**_*k*_ is updated by the first eigenvector of **U**, and the coefficient vector **x**_*k*_^*T*^ is updated by zero padding of the multiplication of the first column of **V** with Δ(1,1).

The above process, SVD on the reconstruction residuals matrix, is applied *K* times to update the codewords each at a time. The details of learning codebook by OMP and* K*-SVD are shown in Algorithm 1.

### 2.4. Middle-Level Feature Extraction for Medical Images

With the prepared training local descriptors **Y** = [**y**_1_, **y**_2_,…, **y**_*N*_], the codebook **D** = [**d**_1_, **d**_2_,…, **d**_*K*_] can be learned using the conventional* K*-means or sparse coding strategy. In order to code the local descriptors **y**_*m*_ ∈ {**y**_1_, **y**_2_,…, **y**_*M*_} from any test image and extract the middle-level feature, we fix the codebook **D** and obtain the coded vector **x**_*m*_ by minimizing the following cost function:

(5) arg minxm ym−Dxm22,s.t. xm0≤αK.

This optimization problem is also implemented by the OMP method, which is called coding strategy and then *M* coded vectors with* K*-dimension for each image can be obtained.

Finally, the *M* coded vectors are aggregated to form a fixed-length feature for image representation by average operator, which is also called pooling procedure. In our application, we apply multiphase CT volumes for FLL retrieval, and thus we extract the codebook-based feature for each phase CT image and directly concatenate all features for multiphase CT representation.

## 3. Experiments and Results

### 3.1. Dataset

**Figure 3:**
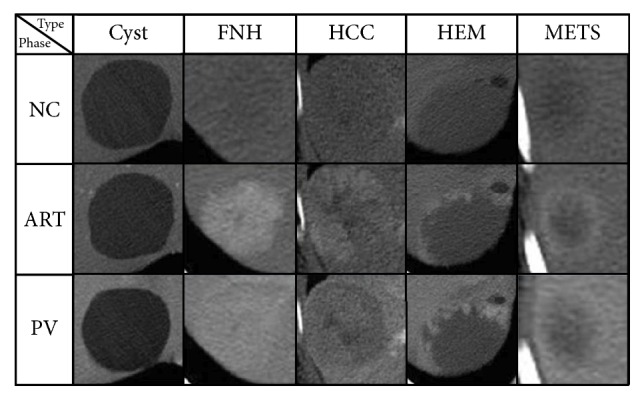
Examples of each lesion type on 3 phases. Rows are images that belong to same contrast phase, while columns are images from the same lesion: cyst, FNH, HCC, HEM, and METS.

The proposed codebook model is used to learn features for retrieval of focal liver lesions (FLLs) from the dataset which we have constructed with the help of radiologists. The dataset consists of 5 types of liver lesions, and there is 137 medical cases in total in the dataset, including 38 lesions in cyst class, 28 cases for both hepatocellular carcinoma (HCC) and hemangioma (HEM), 22 cases in focal nodular hyperplasia (FNH), and 21 liver lesions for metastasis (METS). Due to the fact that most of the medical cases supplied by radiologists contain only three phases (NC, ART, and PV), while only small amount of them have the DL phase, we choose to use only 3 phases for each case for consistency concern. Examples of our dataset are shown in Figure 3.

### 3.2. Intensity Normalization

**Figure 4:**
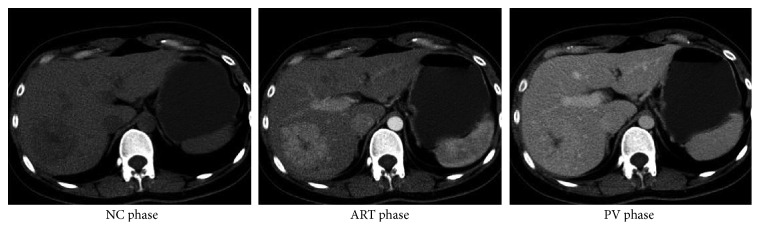
Illustration of intensity inconsistency among different phases.

We found that the intensity distributions of the same patient are different for the three phases, as illustrated in Figure 4. This is caused by the contrast enhancement injection. Though the lesion regions are obviously enhanced, it is a fact that the other tissues, such as health liver parenchyma, are also enhanced at the same time. This breaks down the intensity consistency when combining multiphase data together. Considering this situation, a simple preprocessing technique is employed: the intensities are normalized by the average intensity of the surrounding healthy liver parenchyma. The preprocessing method can not only avoid the inconsistency of intensity distribution among different phases, but also address the problems caused by images captured from different equipment and other intensity-shift problems. The following experiments are all based on the intensity normalized images.

### 3.3. Experiments Settings (Image Representations Based on BoVW)

The image representation based on Bag-of-Visual-Words technique regards an image as a distribution of local descriptors, which can be viewed as discrete visual prototypes. Given a codebook, the occurrence of the descriptors from an image on the codewords is used to represent the image, in a histogram-like style. Since the medical images are commonly taken under standard situations, no meaningful key points or structures appear in a liver lesion in CT images, and the intensity plays an important role in diagnosis. In this work, we use raw patches, which are densely sampled from every pixel in the lesion region, as the local descriptors of a medical image.

Then it is important to learn an effective codebook from the training samples. The simple and efficient algorithm,* K*-means, is widely used in this task for natural images. The proposed codebook learning method based on OMP and* K*-SVD algorithms [18, 19] is used in this study instead of* K*-means.

We first validated the representation accuracy of sparse coding comparing to* K*-means method. In this evaluation, we used 5000 local patches from training ART images and approximated them with* K*-means and sparse coding methods with codebook size 100 for both. Then the reconstruction errors (RE) can be calculated for all the selected patches, and the distributions of the RE of the samples using both methods are plotted in Figure 6. As illustrated in Figure 6, sparse coding method achieves smaller reconstruction errors than using* K*-means method, which means more accurate approximations, and thus more accurate retrieval performance can be expected.

**Figure 5:**
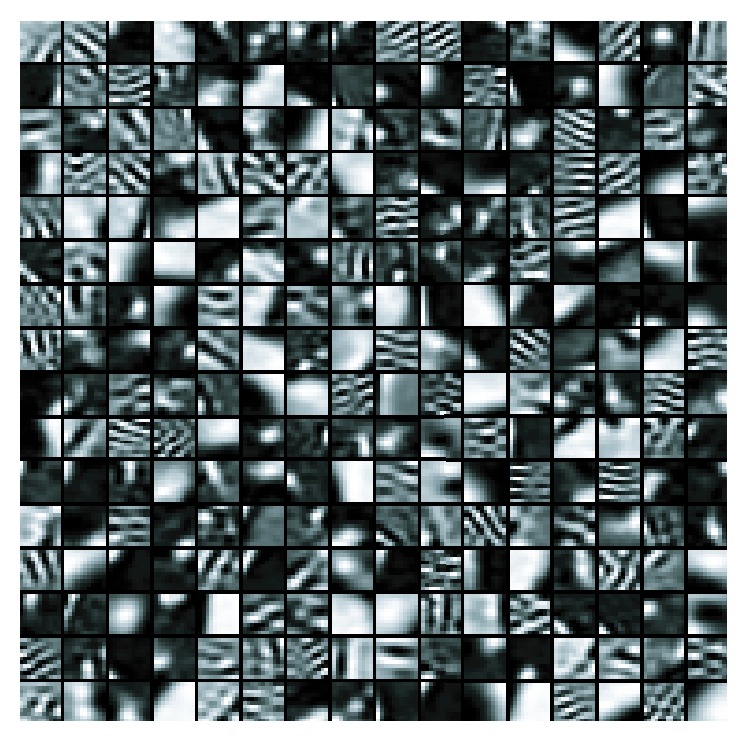
The codebook learned for CT scans on ART phase. The patch size is 11 × 11, and codebook size is 256.

**Figure 6:**
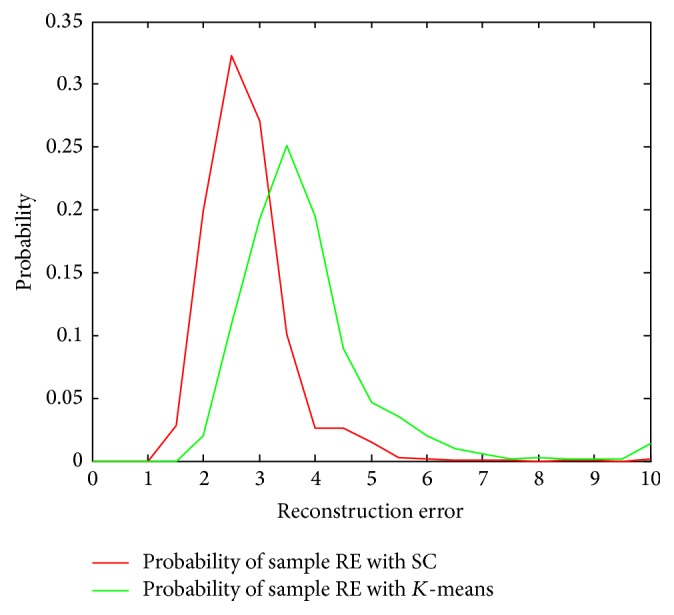
Distribution of sample RE using the proposed method and* K*-means method.

Considering the multiphase medical images, codebooks for each phase are learned separately. Examples of the learned codebook model for ART phase CT scans are shown in Figure 5 and the contrast is enhanced for easy view. With the codebook model for single phase, the retrieval performance will be discussed in the Results section to emphasize the usefulness of contrast enhancement for detection of FLLs. Due to large amount of descriptors which will cost too much time for codebook learning, we randomly select 50000 descriptors from training samples for codebook learning. Since the codebook learning is an unsupervised learning procedure, the selected descriptor number usually does not affect the final performance greatly. Then the coefficients of patches are calculated according to the learned codebooks responding to the image's phase in single-phase condition. The coefficients of patches that belong to the same image are pooled together to form a feature vector to represent the image. In our experiments the mean-pooling technique is used due to its simplicity and efficiency. The feature vectors of images in each phase are concatenated to the final representation of a patient case to engage the multiphase features.

### 3.4. Performance Evaluation

To evaluate the performance of the proposed codebook model for retrieval of FLLs, confusion matrices, Table 1, are built and precision and recall values are calculated correspondingly. Precision versus recall curves [20] are used to represent the precision and recall values, which summarize the pairs of precision and recall pairs when varying the number of retrieved top similar samples.

The leave-one-out cross validation method is used in evaluation. Confusion matrices, as shown in Table 1, are constructed when* t* (1 ⩽ *t* ⩽ *T* − 1) most similar samples are retrieved, where *T* is the total number of medical cases in the dataset. Then precision and recall are calculated correspondingly. A precision versus recall curve is obtained by averaging precision and recall values of all validated samples at position *t* (1 ⩽ *t* ⩽ *T* − 1).

### 3.5. Results

**Figure 7:**
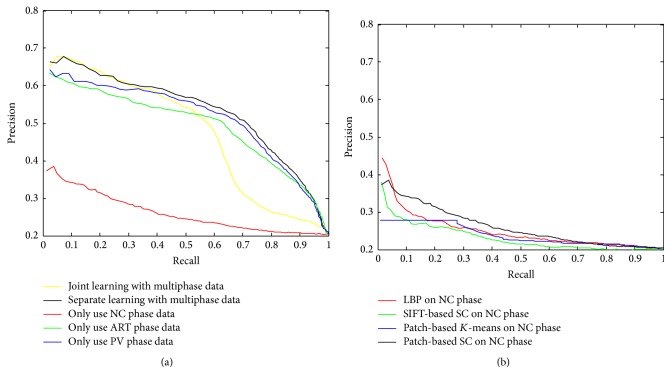
(a) Retrieval performance using single-/multiphase data. (b) Retrieval performance of different methods by using only NC phase data.

Experiments are applied on single-/multiphase medical CT scans. The retrieval performances using single-phase data and multiphase data are shown in Figure 7(a). As shown before in Figure 3, the contrast enhanced phases (ART and PV) show more distinguishable characteristics than the non-contrast enhanced phase (NC). Thus it is reasonable that ART and PV phase have better retrieval performance than NC, while the two contrast enhanced phases have similar performance, as shown in Figure 7(a). The proposed sparse codebook model using multiphase data provides the best performance with the help of temporal information in retrieval of liver lesions. There are two ways to learn codebooks for representation of multiphase data: joint learning and separate learning. Joint learning means that the corresponding local descriptors of data in each phase are combined before learning codebooks. Only one codebook is learned for multiphase data in joint learning. On the other hand, separate learning learns codebooks for each phase and the features of CTs in each phase are concatenated for the final representation of the multiphase data. The compared performance is also given in Figure 7(a). Considering the situation that, in medical practice, the multiphase data are only roughly aligned, and small translations and/or rotations in soft tissues, such as liver, unavoidably exist, which can be caused by factors such as the patient's breath during CT scanning. Because the multiphase data are not exactly registered, the joint pixels from different phases may represent different tissues and thus lead to mismatching combination in the joint learning. As shown in Figure 7(a), the joint learning has comparable performance for a few retrieved cases with separate learning, while the performance reduces fast when more similar cases are retrieved. Since the separate learning outperforms the joint learning, separate learning is used in the rest of the experiments for multiphase data.

Furthermore, the retrieved performance is quiet bad in using the proposed sparse codebook model with the NC phase only. In order to investigate the applicability of the proposed sparse codebook model, we also conducted experiments with state-of-the-art methods on the NC phase only, and the compared retrieval results are provided in Figure 7(b), which illustrated that the proposed patch-based sparse coding (SC) method gets the best performance while all the methods have very poor performance on NC phase data only.

**Figure 8:**
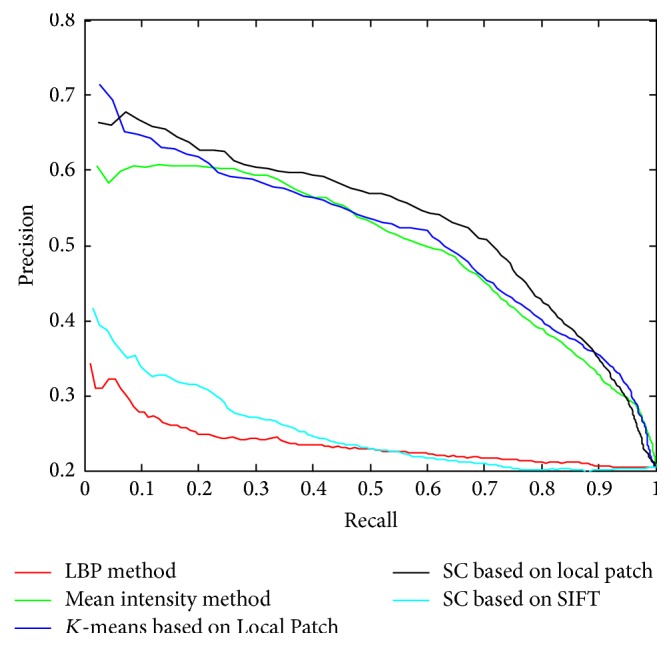
Comparison of the proposed codebook model with state-of-the-art methods.

We compared our method to other alternative methods. We implemented the intensity-based method, which means that we used the mean intensity of the focal liver lesion region to represent the image. Then each medical case is featured by a vector that has 3 elements, one from each phase (NC, ART, and PV). We also realized the codebook learning by* K*-means clustering technique, which refers to* K*-means-based method, with one hundred clustering centroids. In this realization, the training samples are raw patches, which are the same as the training samples in our proposed method. The retrieval performance comparison is shown in Figure 8: the intensity-based method has comparable results which emphasizes the importance of intensity in focal liver lesion recognition; the proposed codebook model has the best performance while the* K*-means-based method only performs slightly better than the intensity-based method.

We have also used the Local Binary Pattern (LBP) to capture the local texture features of focal liver lesions. Each image is represented by a histogram with 256 bins from calculating the LBP patterns. And the medical case is featured by a 768-dimensional vector, which is a concatenation of the three 256-bin histograms for each phase of medical image.

We also used the retrieval accuracy at top *t* retrieved samples to evaluate the performance of different methods.  Table 2 shows the retrieval accuracy of the proposed method; comparing with that of three other methods, when *t* is at the top (*t* = 1,3, 5,10,20), most similar medical cases are retrieved. The* K*-means-based method gets best performance when only one case is retrieved and the proposed sparse codebook model can achieve best performance when more cases are retrieved. The retrieval precisions are very low by using the LBP-based methods, due to the fact that the local texture feature is not discriminative for different types of focal liver lesions.

In addition, we also conducted FLL retrieval experiment with the proposed sparse codebook model using Scale-Invariant Feature Transform (SIFT) as local descriptors, and the result is manifested in Figure 8. Figure 8 shows that the performance of SIFT-based method is very poor and is not acceptable, which explains that retaining all detail structure with local patch is more important than the quantized-based SIFT for the fine-grained medical data representation.

### 3.6. Parameter Optimization

Previous studies suggested that the codebook size and the patch size have significant influence on the performance of image retrieval and categorization for natural images. We evaluated the performance for retrieval of focal liver lesions when varying the patch size, codebook size, and sparsity measure. In previous section, we have proved that retrieval based on multiphase medical data will have the best performance. The following experiments were performed on multiphase CT scans.

#### 3.6.1. Patch Size

**Figure 9:**
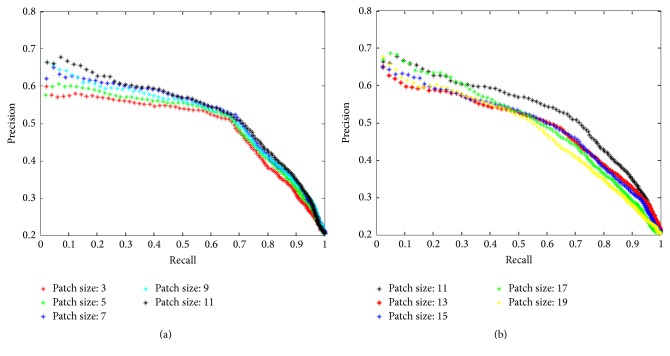
Retrieval performance when varying patch size from 3 to 19.

To evaluate the influence of patch size on the performance of FLLs retrieval, we fixed the other parameters based on our experience and preliminary results (codebook size is set to 100 and sparsity measure is set to 0.1). We have varied the patch size from 3*∗*3 to 19*∗*19 with step size 2, and the results are shown in Figure 9. The retrieval performance will increase as patch size enlarges, Figure 9(a), when using small patch size. This might be explained by the fact that large patches can capture more texture information and thus represent an image more effectively. The performance approaches its optimal at patch size 11*∗*11. When increasing the patch size even further, the performance gets lower, since too large patches will capture tissues from surrounding region, which are regarded as noise and influence the retrieval accuracy. From this evaluation, the patch size will be set to 11*∗*11 in future experiments to achieve the best performance.

#### 3.6.2. Codebook Size

**Figure 10:**
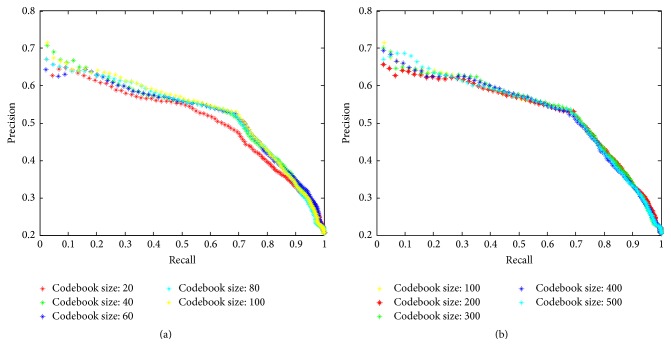
Retrieval performance when varying codebook size.

The codebook size is varied in range from 20 to 100 every 20, when the patch size and sparsity measure are fixed to 11*∗*11 and 0.1, separately. We can see from Figure 10(a) that there is small improvement in the retrieval performance when increasing the codebook size. Similar situation occurs in the performance of ADMM implementation method: no obvious improvement with larger codebook size. We have also explored even larger codebook size, from 100 to 500 every 100, while no better results can be achieved, shown in Figure 10(b). Considering getting a balance between retrieval performance and computation time, the codebook size used in other experiments is determined to be 32.

#### 3.6.3. Sparsity Measure

**Figure 11:**
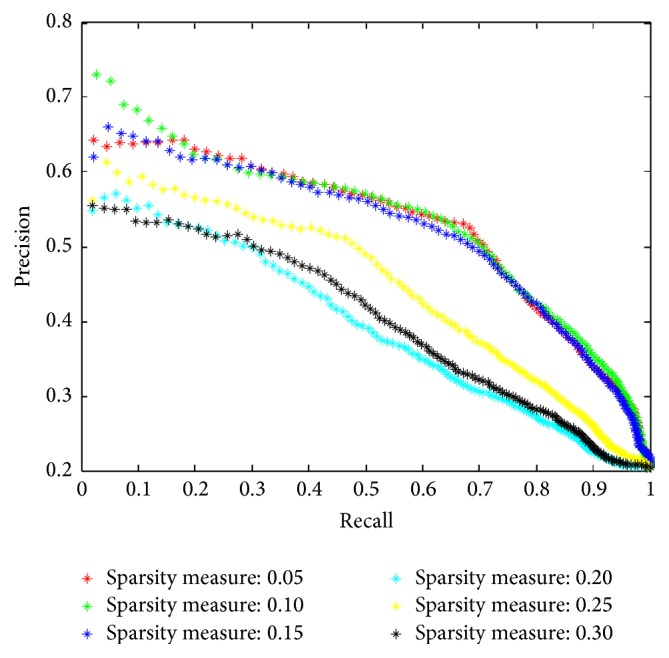
The retrieval performance with various sparsity measures, when patch size is 11*∗*11 and codebook size is 32.

The sparsity measure, *α*, in formula (2), in this work refers to the ratio between the number of nonzero entries of a sparse approximation and the total number of codewords in the learned codebook. *α∗K* controls the maximum number of codewords that can be used for approximation of the input signal; *K* is the size of codebook. We evaluate retrieval performance with different sparsity measure *α*, which varies in {0.05,0.1,0.15,0.2,0.25,0.3}, when patch size is fixed to 11*∗*11 and codebook size is fixed to 100 according to our previous experiments. The experimental results are shown in Figure 11.

### 3.7. Evaluation of ADMM Implementation Method in Sparse Coding

**Figure 12:**
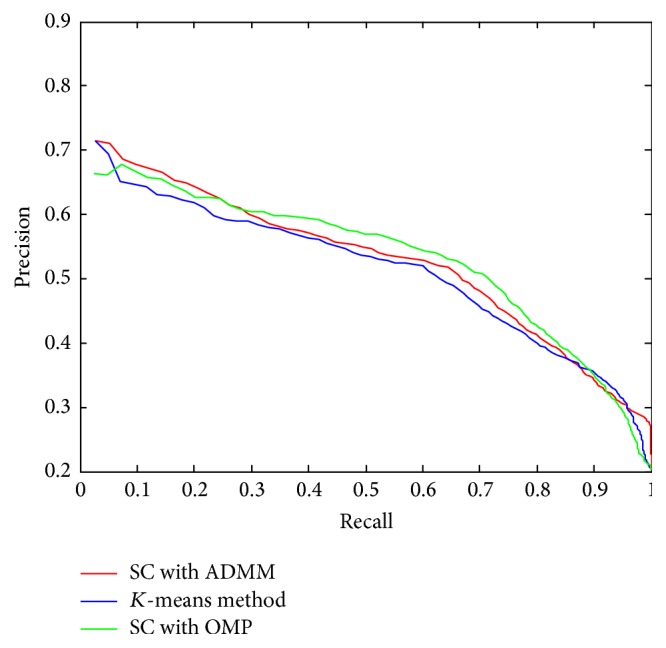
Retrieval performance comparison of ADMM implementation method with the proposed method and* K*-means method.

All the above experiments of our proposed sparse codebook model were conducted with the implementation methods: OMP for coefficient calculation and* K*-SVD for codebook updating. We also implemented the sparse codebook model using ADMM for solving sparse coding optimization problem. The retrieval performance with* K*-means, and our proposed sparse codebook model with* K*-SVD/OMP and ADMM solving methods using codebook size 32 only, respectively, are provided in Figure 12. As shown in Figure 12, the sparse coding method based on either ADMM or* K*-SVD/OMP optimization methods outperforms the* K*-means method. The sparse coding with ADMM implementation is better than that with* K*-SVD/OMP when only several similar cases to the query case are retrieved, while the SC with* K*-SVD/OMP performs better when more similar cases are retrieved.

**Figure 13:**
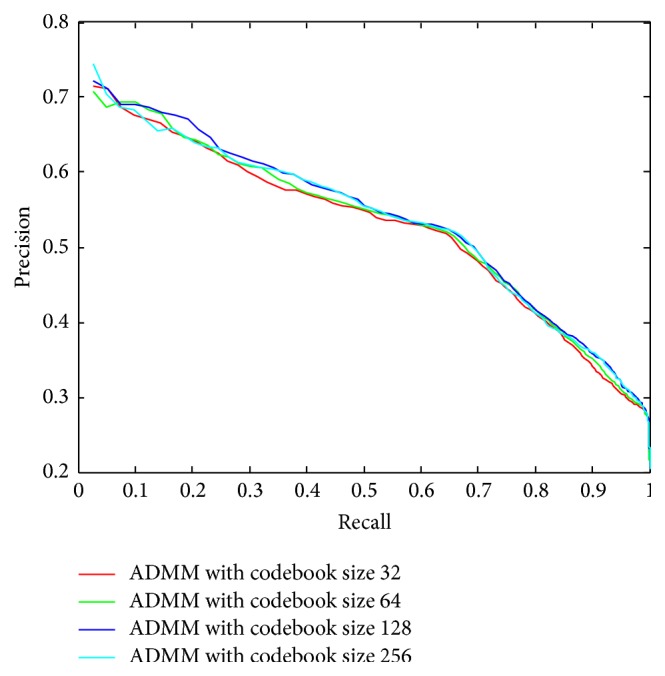
Retrieval performance of ADMM implementation method with different codebook sizes.

We have also evaluated the performance of the ADMM method with different codebook sizes, as shown in Figure 13. The retrieval performance improvement is not obvious when increasing the codebook size. Considering the time efficiency, the codebook size used in performance comparison with other methods, Figure 12, is determined to be 32.

### 3.8. Illustration of Top Retrieved Results

**Figure 14:**
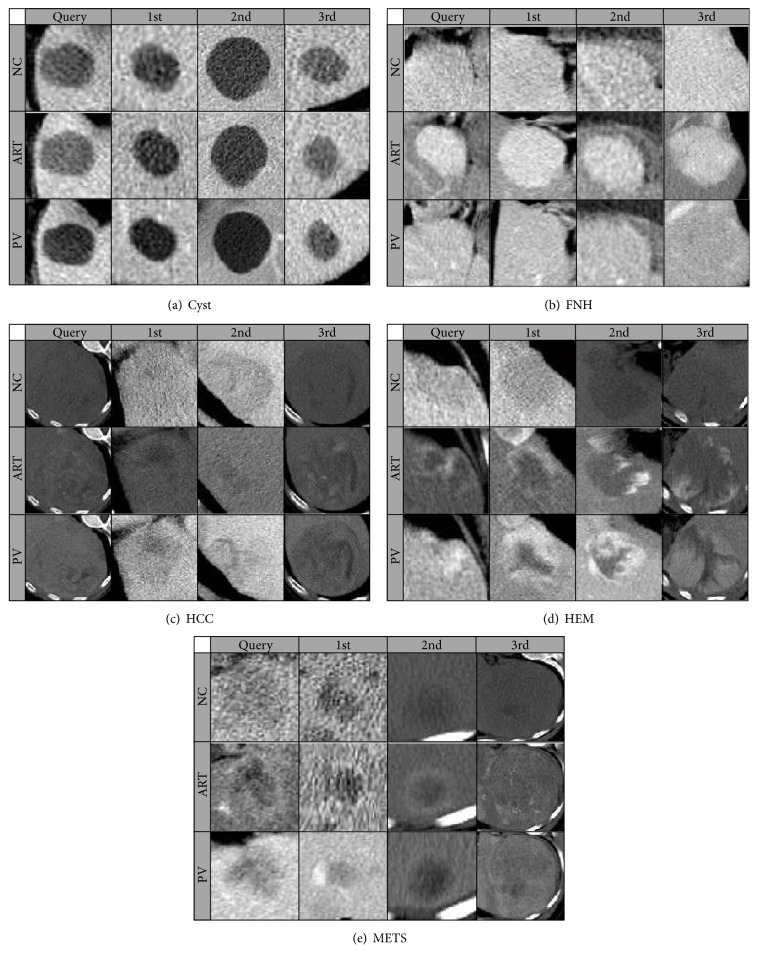
Illustration of retrieval results (top three retrieved examples) for one example from each class.

The top three retrieved samples for one example from each focal liver lesion class are shown in Figure 14. The retrieval is performed using the proposed codebook model with parameters that are all optimal, which have been proved in previous section. The three rows of images show the representative slices of the corresponding focal liver lesion on different phases. The first columns are the query samples while the following three columns are the top three retrieved samples using the leave-one-out cross validation. We can see that the retrieved samples have very similar appearance with query samples. What is more, similar enhancement characters are shown between the query and retrieved samples. However, there are some cases where the top retrieval sample does not belong to the same class as the query sample, for example, the 3rd retrieved sample belongs to HCC for the query example from METS class.

## 4. Conclusion

In this work we present a codebook model for retrieval of focal liver lesions using three-phase contrast enhanced CT images. The proposed method is evaluated on a dataset with 137 medical cases comprising five types of focal liver lesions. The leave-one-out cross validation is applied in construction of confusion matrices for retrieval of each medical case. Codebook is learned for medical cases in each phase separately. And thus the middle-level features based on the proposed sparse codebook model can be extracted from each phase data and the concatenated vector of all phase features is used as the representation of the multiphase medical data. Experimental results show that retrieval based on multiphase data achieved best performance while using only one contrast enhanced CT image can also get reasonable results. In future works, we will extend the linear analysis to multilinear analysis of the medical data to capture core information. Since the vectorization of local descriptors is unavoidable in linear analysis, the spatial structure is always destroyed. We want to apply the tensor technique which analyzes the input signals without unfolding and thus keep the structure for better representation of high-dimensional medical data. With improved accuracy in future works, we intend to develop a content-based image retrieval system to assist in medical education and clinical practice.

## Figures and Tables

**Algorithm 1:**
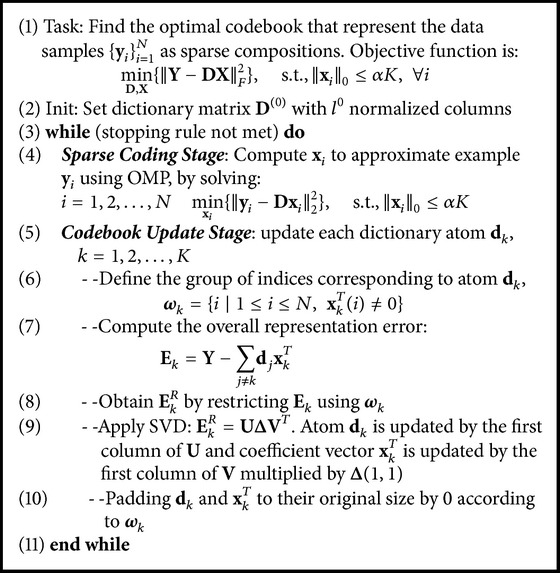
Codebook learning via OMP and *K*-SVD.

**Table 1:** Construction of confusion matrix.

	Actual positive	Actual negative
Predicted positive	TP	FP
Predicted negative	FN	TN

Precision = TP/(TP + FP).

Recall = TP/(TP + FN).

**Table 2:** Retrieval accuracy of different methods at various most similar numbers of cases being retrieved.

Methods	Top 1	Top 3	Top 5	Top 10	Top 20
Proposed method	0.66	0.68	0.65	0.62	0.59
Mean intensity method	0.61	0.6	0.6	0.6	0.56
*K*-means based on local patch	0.72	0.65	0.64	0.61	0.56
LBP method	0.34	0.31	0.32	0.28	0.25
